# Methods and Applications in Proteins and RNAs

**DOI:** 10.3390/life13030672

**Published:** 2023-03-01

**Authors:** Haoquan Liu, Zhou Gong, Yunjie Zhao

**Affiliations:** 1 Department of Physics, Institute of Biophysics, Central China Normal University, Wuhan 430079, China; 2 State Key Laboratory of Magnetic Resonance and Atomic Molecular Physics, Innovation Academy for Precision Measurement Science and Technology, Chinese Academy of Sciences, Wuhan 430071, China

Proteins and RNAs are primary biomolecules that are involved in most biological processes. Coding RNAs are responsible for gene expression and protein synthesis, whereas non-coding RNAs are involved in various biological regulations within cells [1]. Proteins perform essential biological functions, including catalyzing biochemical reactions, repairing DNA, providing structural support, etc. The function of a biomolecule depends on its tertiary structure. Rapid advancements in techniques highlight the race to understand the functions and mechanisms of disease at a molecular level.

In recent decades, several experimental techniques have been developed to determine the structures of proteins and RNAs. X-rays incident on crystal interiors and then diffract in different specific directions. X-ray crystallography was the most effective experimental technique to determine the structure from a three-dimensional electron density map in recent decades [2]. NMR analyzes the structure and dynamics from various data such as chemical shift, relaxation parameters, distance, etc., produced from multi-dimensional spectrums [3]. Moreover, NMR can study solvent and membrane environments’ structural and dynamic behaviors [4]. Cryo-electron microscopy displays radiation-sensitive specimens in a transmission electron microscope under cryogenic conditions [5]. Recently, single-particle cryo-EM has attracted considerable buzz in structural biology for solving protein machines. The Protein Data Bank (PDB) currently contains 201,515 entries (before 17 February 2023). The proportions of structures determined by X-ray crystallography, NMR, and cryo-EM are 85.89%, 6.86%, and 6.98%, respectively. However, there are still some limitations to the above experimental methods. The sample must be crystallizable for X-ray crystallography. Second, NMR can only analyze small structures with molecular weights less than 50kDa. It remains a challenge to obtain high-resolution structures of molecules smaller than 200kDa using single-particle cryo-EM [6]. In addition, all the above structural biology experimental methods have requirements for the purity and stability of the sample. There are still billions of sequence-known biomolecules that need to be determined.

Several computational methods have been developed to predict protein and RNA tertiary structures. For example, Alphafold2 outperforms other traditional methods in the 14th and 15th Critical Assessment of protein Structure Prediction (CASP14 and CASP15) and triggered a revolution in the science community [7,8,9]. For the RNA study, Zhao et al. illustrated 3dRNA which utilized the smallest secondary structure elements to predict RNA tertiary structures automatically [10]. Zhou et al. proposed FebRNA to build tertiary RNA structures through coarse-grained fragment assembly [11]. Traditional methods use knowledge-based statistical potential or force fields to evaluate RNA structures. RNA3DCNN [12] and ARES [13] use deep learning-based frameworks to identify the near-native structures and improve the RNA tertiary structure prediction. However, complex structure prediction remains one challenging and critical issue. Huang et al. proposed 3dRPC to predict RNA-protein complexes by combining the RPDOCK and DECK-RP functions [14]. Yan et al. developed HDOCK for protein–protein docking using template-based modeling and a free docking strategy [15].

There are many cutting-edge techniques to solve challenging problems in RNA and protein studies [16,17]. This Special Issue intends to encourage researchers from multidisciplinary backgrounds to share their expertise in diverse aspects of methods and applications. We hope this topic will further promote scientific discoveries in the health-related scientific community.
